# Organ Repair and Regeneration During *Ex Situ* Dynamic Preservation: The Future is Nano

**DOI:** 10.3389/ti.2023.11947

**Published:** 2023-11-10

**Authors:** Nicholas Gilbo, Joris Blondeel, Jacques Pirenne, Renato Romagnoli, Giovanni Camussi, Diethard Monbaliu

**Affiliations:** ^1^ Laboratory of Abdominal Transplantation, Department of Microbiology, Immunology and Transplantation, Faculty of Medicine, KU Leuven, Leuven, Belgium; ^2^ University Hospital of Liège, Liège, Belgium; ^3^ University Hospitals Leuven, Leuven, Belgium; ^4^ General Surgery 2U–Liver Transplant Unit, A.O.U. Città della Salute e della Scienza di Torino, University of Turin, Turin, Italy; ^5^ Dipartimento di Chirurgia Generale e Specialistica, Azienda Ospedaliero Universitaria Città della Salute e della Scienza di Torino, Turin, Italy; ^6^ Department of Medical Sciences, School of Medicine, University of Turin, Turin, Italy; ^7^ Molecular Biotechnology Center, Department of Molecular Biotechnology and Health Sciences, School of Medicine, University of Turin, Torino, Italy

**Keywords:** organ repair, organ regeneration, machine perfusion, dynamic preservation, organ preservation, mesenchymal stromal cell, extracellular vesicles

## Abstract

Organ preservation and assessment with machine perfusion (MP) has provided transplant physicians with the ability to evaluate and select grafts suitable for transplantation. Nevertheless, the discard of organs considered too damaged still sustains the imbalance between donor organs supply and demands. Therefore, there is the pressing clinical need for strategies to repair and/or regenerate organs before transplantation, and MP is uniquely positioned to satisfy this need. The systemic administration of mesenchymal stromal cells (MSC) was shown to reduce ischemia-reperfusion injury in pre-clinical organ transplant models but could not be reproduced in clinical transplantation, largely because of inefficient cell delivery. The administration of MSC during MP is one strategy that recently gained much attention as an alternative delivery method to target MSC directly to the donor organ. However, careful reinterpretation of preliminary results reveals that this approach is equally limited by a suboptimal delivery of short-lived MSC to the target organ. In contrast, the use of MSC secretome and/or extracellular vesicles therapy during MP seems to be more efficient in harnessing MSC properties during MP. In this mini review we speculate on the future of the novel niche of *ex situ* organ repair and regeneration before transplantation.

## Introduction

The field of organ preservation for transplantation has undergone significant changes due to the increasing use of grafts from high-risk donors. The need for improved preservation of these organs has prompted a progressive shift from static cold storage to dynamic organ preservation strategies, also known as machine perfusion (MP). Dynamic organ preservation strategies have also moved the field at the intersection with regenerative medicine as they provide a platform for repairing and regenerating organs before transplantation [1]. However, prolonged *ex situ* preservation for multiple days is likely required to achieve clinically meaningful organ repair and regeneration during MP. Recent advancements in liver normothermic-MP (NMP), which allows for the preservation of the liver for up to 1 week [2], suggest that this may soon be attainable for all other transplantable organs.

To this end, several interventions during MP have been proposed, including cell therapy, pharmacological agents, gene modulation and editing, and nanoparticles [3]. Whereas most of these strategies are still in early stages of investigation, numerous pre-clinical studies have shown that the systemic administration of mesenchymal stromal cells (MSC) reduces ischemia-reperfusion injury (IRI) during organ transplantation [4]. MSC suppress the inflammatory response, downregulate innate and adaptive immunity, and promote organ regeneration, thereby interfering with the major pathophysiological events of IRI in transplantable organs [4]. However, the clinical application of MSC systemic treatment during organ transplantation has failed to replicate these results [5], one of the major putative cause being the inefficient delivery of MSC to the target organ. Therefore, MSC administration during MP has gained interest as an alternative method to deliver the cells directly to an organ and circumvent the shortcomings of systemic administration. Nevertheless, the efficiency of this approach in delivering MSC-therapy to organs for transplantation remains underinvestigated. In this narrative minireview, we summarize results and limitations of MSC-therapy during MP based on available evidence from published studies. To select these studies, we utilized a systematic literature search approach, a rigorous method for minimizing biases during evidence selection (see Supplementary Material for additional method information). Additionally, we hypothesize a path towards a cell-free future for *ex situ* organ repair and regeneration.

## Novel Delivery Method, Same Shortcomings

Unlike preclinical transplant models of MSC-therapy, clinical studies have failed to show significant benefits from systemic MSC administration on post-transplant IRI [6, 7]. This was ascribed to inefficient and off-target delivery of cells to the graft [5]. Systemic infusion of MSC are short-lived as the cells are primarily sequestered in the lungs and eliminated by resident monocytes [5]. Administration of stem cells after organ transplantation has also been associated with a pro-inflammatory effect, which further damages the graft [8]. Lastly, calcineurin inhibitors suppress the immunomodulatory properties of MSC *in vitro* [9]. To overcome these hurdles, it was proposed to deliver the cells directly to an organ during MP, before the full extent of IRI events has occurred and interference with immunosuppressive agents can take place.

**FIGURE 1:**
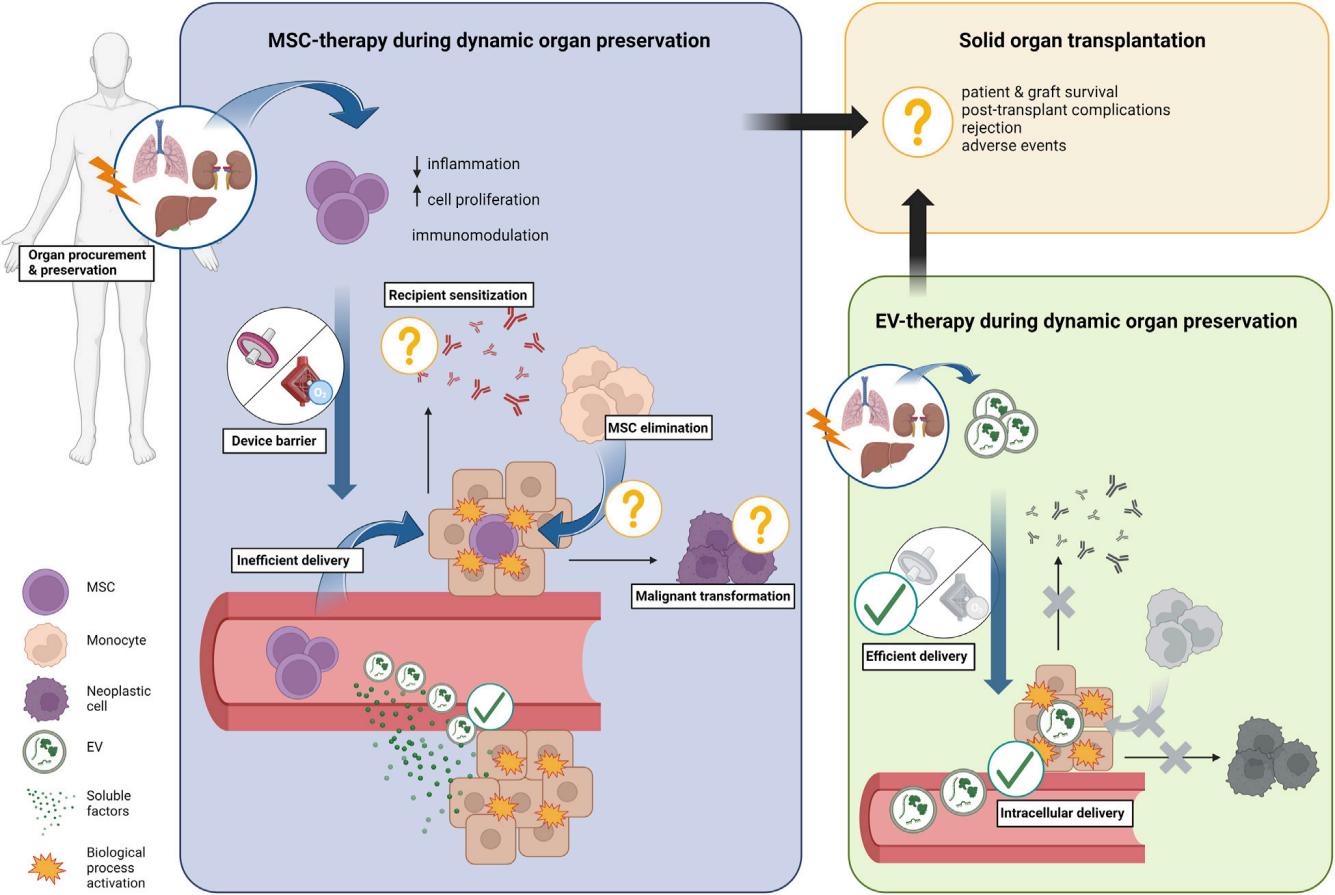
Overview of current knowledge on cell therapy delivery during *ex situ* dynamic preservation. Mesenchymal stromal cells (MSC) are perfect candidates for organ repair and regeneration during *ex situ* dynamic organ preservation due to their anti-inflammatory, regenerative, and immunomodulatory properties. However, a “device barrier” created by components of the perfusion circuit (i.e., oxygenator(s) and filter(s)) sequester the cells from the circulating perfusate, with a bottleneck effect on the number of cells that effectively reach the parenchyma during perfusion. Additionally, migration of stem cells out of the vascular space is infrequent, only few cells are usually observed in the parenchymal space at histology, and parenchymal retention rate of stem cell is rather low. Furthermore, monocytes may actively eliminate successfully engrafted stem cells, similarly to what has been previously observed in pre-clinical studies. Because secretion of soluble factors has been observed during perfusion, it is plausible that the biological effects of stem cells are mostly dependent on paracrine mediators, extracellular vesicles (EV) included. EV delivery during *ex situ* dynamic organ preservation has been shown to circumvent the “device barrier” while intracellular uptake of EV has been demonstrated during lung and liver perfusion specifically, resulting in significant anti-inflammatory and regenerative effects. Therefore, EV therapy may be more effective than MSC therapy in promoting organ repair and regeneration during *ex situ* dynamic organ preservation because of more efficient therapy delivery. Next to a more efficient therapy delivery, cell-free therapy with EV prevents resident monocytes activation and eliminates the risk of malignant transformation and recipient sensitization, which cannot be excluded when allogenic stem cells are administered.

However, the currently available pre-clinical evidence shows that MSC delivery during MP presents shortcomings similar to those of systemic therapy, as summarized in Table 1 and depicted in Figure 1. A significant proportion of MSC injected through the vascular cannula during MP are eliminated by a “device barrier,” constituted by oxygenator(s) and filter(s), which remove the cells from the perfusate similarly to the “lung barrier” phenomenon in systemic MSC-therapy. In a porcine kidney study, Pool et al. demonstrated that 90% of infused MSC are eliminated from the perfusate in a NMP circuit operated without the organ, and that only a few MSC were retained after the first passage through the kidney [10]. In a porcine lung NMP model, Mordant et al. found MSC sequestered in the leukocyte filter [11]. Similarly, Laing et al. did not observe any cell in left hepatic segments after selectively delivering multipotent adult progenitor cells (MAPc) to the right hemi-liver during NMP of discarded human grafts [12]. These results indicate that, at best, only a (small) fraction of MSC is effectively retained in the perfused organ. Additionally, biodistribution studies have shown that MSC have an inhomogeneous distribution in otherwise well-perfused kidney [10] and liver [13] grafts. In accordance with this, histological studies have shown that <10 cells per high-power field are found outside of the vascular space during MP of the liver [12, 13], lung [11], and kidney [14, 15]. Although in the study by Pool et al. increasing the dose of MSC increased the number of cells observed at histology, a dose of MSC far exceeding the previously suggested range for MSC systemic therapy was needed to visualize the cells in the glomeruli of porcine kidneys [10]. Lastly, five other studies reported that MSC did not leave the perfusate or migrate out of the vascular lumen during MP of human and porcine kidneys [16] and rat livers [17–20] (Table 1).

**TABLE 1:** Summary of findings of studies investigating stem cell therapy delivery during *ex situ* dynamic organ preservation identified after systematic search of the literature (details in Supplementary Material). Results from preliminary studies investigating extracellular vesicle therapy during machine perfusion of transplantable organs are also summarized.

Studies investigating mesenchymal stromal cell delivery during dynamic organ preservation													
Study	Subject	Organ	Organ transplant	Type and duration MP	MSC type	MSC dose	MSC paracrine activity during MP	Device barrier	MSC migration from vascular space	MSC engraftment^a^	MSC Viability	MSC therapeutic effect	Effect without engraftment
[36]	Human	Lungs (discarded)	No	Normothermic, 4 h	Human	5*10^6^	NA	NA	NA	NA	NA	↑ alveolar fluid clearance	NA
					BM-MSC								
[8]	Human	Lungs (discarded)	No	Normothermic, 4 h	MAPc	10^7^	NA	NA	NA	NA	NA	↓ BAL cellularity & histological inflammation	NA
[9]	Pig	Lungs	No	Normothermic, max 12 h	Human	50*10^6^	NA	Yes, MSC trapped in filters	Yes, some cells in the lumen at histology	Yes,<10 cells/HPF	NA	↓ IL-8 perfusate concentration	NA
					UC-MSC	150*10^6^							
						300*10^6^							
[10]	Rat	Kidneys	No	Hypothermic, 4 h	Rat	3*10^6^	NA	NA	Yes	Yes,<10 cells/HPF	NA	↓ severity histological damage	NA
					BM-MSC								
[37]	Pig	Lungs	No	Normothermic, 6 h	MAPc	150*10^6^	No	NA	No	No	NA	No significant therapeutic effect	NA
[34]	Mouse	Lungs	No	Normothermic, 1 h	Human	3*10^6^	NA	NA	NA	NA	NA	↑ compliance	NA
					UC-MSC							↓ inflammation, neutrophil infiltration & oedema	
[38]	Rat	Liver	No	Normothermic, 2 h	Swine	0.2*10^6^	NA	NA	NA	NA	NA	No significant therapeutic effect	NA
					AD-MSC	10^6^							
[11]	Human	Kidneys (discarded)	No	Sub-normothermic, 24 h	Not specified	25*10^6^	Yes	No	No, 95% MSC still circulating at the end of MP	No	NA	↑ renal cell proliferation & tissue regeneration	Yes
						50*10^6^							
						75*10^6^							
						1*10^8^							
						2*10^8^							
[12]	Pig	Kidneys	No	Normothermic, 7 h	Human	1*10^5^	NA	Yes, Inhomogeneous distribution in well perfused kidneys	No	No	Disintegrated MSC in colonized glomeruli	Study investigating feasibility and biodistribution	NA
					AD-MSC &	1*10^6^							
					BM-MSC	1*10^7^							
[13]	Pig	Lungs	Yes, f-up 4 h	Normothermic, 12 h	Human	50*10^6^/Kg	Yes	NA	Yes, Unspecified proportion of MSC remained in the lumen	Yes, alveolar interstitium	Yes, indirect evidence based on production of human cytokines	During MP: ↓ apoptosis & perfusate concentration of IL-18 and IFNγ, ↓ peak airways pressure	NA
					UC-MSC							Post-transplant: ↓ oedema & severity histological injury, f-up limited to 4 h	
[14]	Human	Kidneys (discarded)	No	Normothermic, 7 h	MAPc	50*10^6^	Yes	No	Yes, Unspecified proportion of MSC kept circulating at the end of MP	Yes, glomeruli in the cortex, peritubular space in the medulla	21% of circulating MSC were viable	↑ urinary output & medullar flow	NA
												↓ urinary concentration NGAL & perfusate concentration IL-1β	
												↑ perfusate concentration IL-10	
[15]	Human	Liver	No	Normothermic, 6 h	MAPc	50*10^6^	Yes	Yes, MSC infused via left hepatic vessels did not reach right segments	Yes, only if infused via the hepatic artery	Yes, only if infused via the hepatic artery	NA	↓ perfusate concentration pro-inflammatory cytokines	Yes
												↑ perfusate concentration anti-inflammatory cytokines	
[16], [39]	Rat	Liver	No	Normothermic, 8 h	Rat	1–3*10^7^	NA	NA	No	No	NA	↓ perfusate AST/ALT and severity histological damage	Yes
					BM-MSC							↓ mitochondrial injury	
[17]	Pig	Liver	No	Hypothermic for MSC delivery, 30 min	Human	5*10^6^	Yes	Yes, inhomogeneous distribution in well perfused livers	Yes	Yes	Yes, indirect evidence based on production of human cytokines	Study investigating feasibility and biodistribution	NA
				Normothermic for functional assessment, 4 h	BM-MSC	1*10^7^							
[12]	Pig	Kidneys	No	Normothermic, 7 h	Human	1*10^7^	Yes	NA	NA	NA		No significant therapeutic effect	NA
					AD-MSC & BM-MSC								
[18] ^b^	Pig	Kidneys	Yes, f-up 14 days	Normothermic, 4 h	Human	1*10^7^	NA	NA	NA	Yes, Y human chromosome detected in parenchyma but circa 20-fold ↓ 14 days post-transplant	NA	No safety concern during perfusion, No significant post-transplant therapeutic effect	NA
					AD-MSC								
[19]	Rat	Liver	Yes, f-up 14 days	Normothermic, 4 h	Rat	1*10^7^	NA	NA	No	No	NA	↓ post-transplant AST/ALT release & acute cellular rejection	Yes
					BM-MSC^c^								
[20]	Rat	Liver	No	Normothermic, 6 h	Rat	1*10^7^	NA	NA	No	No	NA	↓ severity of ferroptosis & perfusate AST/ALT concentration	Yes
					BM-MSC	3*10^7^							
[21]	Rat	Liver	Yes, f-up 14 days	Normothermic, 4 h	Rat	1–3*10^7^	NA	NA	NA	Unspecified location in the hepatic tissue	NA	During MP: ↑ proliferation cholangiocyte extrahepatic bile duct and preservation of epithelial lining	NA
					BM-MSC^c^							Post-transplant: ↓ AST/ALT/GGT/bili 7 days post-transplant; ↑ proliferation & ↓ apoptosis peribiliary glands	

Studies investigating extracellular vesicles delivery during dynamic organ preservation									
Study	Subject	Organ	Organ transplant	Type and duration MP	Source of EV	EV dose	EV uptake confirmed	EV therapeutic effect	Compared to MSC
[22]	Human	Lungs (discarded)	No	Normothermic, 6 h	Human BM-MSC	100–200 μL	*In vitro* only (human alveolar epithelial type 2 cell line)	↑ alveolar fluid clearance & ↓ oedema and weight gain, ↑ compliance	No
[10]	Rat	Kidneys	No	Hypothermic, 4 h	Rat BM-MSC	Concentration not reported, EV released by 3*10^6^ cells	NA	↓ perfusate LDH and MDA, ↑ glucose metabolism,↓ severity histological damage	Yes, magnitude of effects of EV > MSC
[34]	Mouse	Lungs	No	Normothermic, 1 h	Human UC-MSC	Concentration not reported, EV released by 3*10^6^ cells	NA	↑ compliance, ↓ inflammation, neutrophil infiltration & oedema	Yes, magnitude of effects of EV = MSC
[23]	Rat	Liver	No	Normothermic, 4 h	Human liver stem-like cells	5*10^8^ EV/g of liver	Yes, intracellular localization in hepatocytes	↓ perfusate AST & severity histological damage	No
[24]	Rat	Lungs	No	Normothermic, 3 h	Human BM-MSC	24.56 ± 5.53 *10^10^ EV/mL, 5 mL were administered	Yes, intracellular localization in alveolar cells	↓ total vascular resistance, ↑ glucose metabolism and tissue content of ATP	No
[25]	Rat	Liver	No	Normothermic, 6 h	Human liver stem-like cells	5*10^8^ EV/g of liver	Yes, intracellular localization in hepatocytes	↓ perfusate AST/ALT & ↑ bile excretion, ↓ necrosis & ↑ hepatocellular proliferation	No
						25*10^8^ EV/g of liver			
[26]	Human	Kidneys (discarded)	No	Hypothermic, 4 h	Human BM-MSC	28.5*10^9	NA	↓ apoptosis & ↑ tubular cells proliferation, ↓ mitochondrial injury	No

^a^ Refers to the visualization of MSC between parenchymal cells (outside of the vascular lining) at histology. When available, the estimated cellular concentration is reported.

^b^ In this study, porcine kidneys underwent 14 h preservation with hypothermic oxygenated MP, followed by 4 h of normothermic MP with or without MSC infusion.

^c^ In these studies, MSC were modified to overexpress the enzyme heme oxygenase 1.

Abbreviations: AD-MSC, adipose-derived mesenchymal stem cells; ALT, alanine transaminase; AST, aspartate transaminase; ATP, adenosine triphosphate; BAL, bronchoalveolar lavage; BM-MSC, bone marrow-derived mesenchymal stem cells; EV, extracellular vesicles; HPF, high-power field; IFNγ, interferone gamma; IL-1β, interleukin 1 beta; IL-8, interleukin 8; IL-10, interleukin 10; IL-18, interleukin 18; LDH, lactate dehydrogenase; MAPc, multipotent adult progenitor cells; MDA, malondialdehyde; MP, machine perfusion; NGAL, neutrophil gelatinase-associated lipocalin; UC-MSC, umbilical cord-derived mesenchymal stem cells.

NA, or not applicable, is assigned when a manuscript reported insufficient details for accurate evaluation.

Next to the “device barrier” and low cell retention rates, there are also indications that MSC infused during MP are short-lived, likely due to factors such as mechanical trauma, perfusate toxicity, or phagocytosis by resident monocytes. Pool et al. consistently observed disintegrated MSC in porcine glomeruli colonized by stem cells [10], whereas Thompson et al. reported that at the end of NMP of discarded human kidneys only 21% of the MAPc still circulating in the perfusate were viable (Table 1) [15]. Research has shown that, compared to standard culturing medium, suspending MSC in a standard red blood cells-based MP perfusate reduces significantly their survival and adherence to endothelial cells [21]. Additionally, because monocytes were already shown to phagocyte MSC [5], it is plausible that resident monocytes and/or passenger leukocytes will eliminate MSC during MP (Figure 1). However, to date this phenomenon has not been investigated yet.

Despite the low cell retention rates, there are indications of significant anti-inflammatory [15, 22], immunomodulatory [18] and pro-regenerative [16, 20] effects of MSC-therapy during MP (Table 1). Nevertheless, the clinical relevance and durability remain unclear as the few studies that transplanted MSC-treated grafts have only reported short-term follow-ups with contrasting results (Table 1). Rat livers were transplanted after MSC-therapy during MP, showing significant improvement of survival and reduction of the incidence of acute cellular rejection at 14 days post-transplant [18]. Porcine lungs treated with MSC were transplanted and followed up for 4 h after reperfusion, showing reduction of pulmonary oedema and severity of histological injury [23]. In contrast, porcine kidneys that were transplanted after MSC-therapy during NMP showed no relevant therapeutic effect within 14 days after transplantation [24].

Hence, while a direct comparison is lacking, available pre-clinical evidence indicates that MSC-therapy during MP presents similar shortcomings and may not be more effective than MSC systemic therapy in delivering the cells to the graft. Furthermore, allogeneic MSC-therapy during MP does not eliminate the potential for recipient allo-sensitization to cell donor antigens [6, 25] or malignant transformation of (the few) successfully engrafted cells. These are two potential complications that cannot be ruled out when allogeneic stem cells are administered to patients who will receive immunosuppressants after transplantation.

## Cell-free Organ Repair and Regeneration During *Ex Situ* Dynamic Preservation

While MSC administration during MP does not have high efficiency in cell delivery, the anti-inflammatory [12], immunomodulatory [18], and regenerative [16, 20] effects of MSC, as well as significant reduction in the severity of graft injury [14, 17, 19, 20] have been observed during perfusion. These effects were observed even when MSC remained suspended in the perfusate, did not migrate out of blood vessels, or did not survive (Table 1). Most strikingly, Brasile et al. found that renal cell proliferation was significantly enhanced in perfused kidneys despite the fact that 95% of MSC did not migrate in the renal tissue but remained in the perfusate for 24 h [16]. This effect was attributed to the release of growth factors by MSC [16]. Several other MP studies reported that MSC actively secrete soluble and paracrine factors in the perfusate (Table 1) [12, 13, 15, 23, 26]. The frequent observation that MSC have significant detectable effects during MP even when no direct contact between MSC and parenchymal cells has taken place, and that MSC secrete paracrine mediators during MP, strongly suggest that their effects rely mostly on soluble factors and paracrine mediators, such as growth factors, cytokines, chemokines, and extracellular vesicles (EV). This implies that the biological properties of MSC beneficial against organ IRI could be harnessed during MP with a cell-free therapy consisting of MSC secretome and/or purified EV [4, 5].

EV are nano-sized particles released by every (stem) cell. As they contain genetic information, growth factors, and signal transduction molecules [4], they play an important role in (stem) cell-mediated regulation of homeostasis and orchestration of tissue regeneration [27]. Upon internalization by neighboring or distant target cells, EV release their biological active cargo and induce epigenetic modifications of target cell biology, mediating the biological effects of the parent stem cell. During *ex situ* dynamic organ preservation, cell-free therapy with concentrated stem cell-derived EV has already shown encouraging results (Table 1). Studies have already demonstrated that EV are taken up by alveolar cells and hepatocytes during perfusion in rodent models of NMP of freshly procured lungs [28] and livers [29], resulting in significant improvements in pulmonary metabolism and adenosine triphosphate content [28], as well as reduction in transaminases and severity of histological injury during perfusion [29]. Additionally, in the study by De Stefano et al., EV from human liver stem-like cells reduced hepatocellular injury and increased cell proliferation during NMP of rat livers that suffered 60 min warm ischemic injury [30]. Gennai et al. showed that EV-therapy during NMP of discarded human lungs significantly improved alveolar fluid clearance, reducing inflammation and pulmonary oedema [31]. Gregorini et al. delivered MSC-EV during hypothermic-MP of rat kidneys, showing a significant reduction in markers of renal injury and oxidative stress [14]. The same group reported similar observations with EV-therapy during hypothermic-MP of discarded human kidneys [32]. If replicated, these findings would indicate that there may be an additional window of opportunity to deliver cell-free therapy during hypothermic dynamic organ preservation. However, transplantation of grafts treated with EV during MP has not been attempted yet, and future studies should focus on testing the hypothesis that EV-therapy at the time of MP affects post-transplant outcomes.

Hypothetically, cell-free therapy during MP could also avail of the delivery of MSC secretome, which contains both soluble factors and EV. To our knowledge, this therapeutic option has not yet been investigated.

## Discussion

### The Future is Nano

Dynamic organ preservation strategies have entered the clinical arena and are expected to improve the preservation of high-risk organs. MSC-therapy during MP was proposed as an approach to repair high-risk grafts that are deemed too damaged and render them suitable for transplantation [1, 3]. However, there is sufficient accumulated evidence to conclude that MSC are short-lived during MP and poorly delivered to the target organ, similarly to systemic MSC-therapy, while the inherent risks of recipient’s sensitization [6] and malignant transformation remain. Therefore, although cell therapy may still play a role for instance in the recellularization of human organ scaffolds, alternative strategies for repairing and regenerating organs *ex situ* should be investigated in the future.

MSC-derived cell-free therapy during MP has several advantages and circumvent the shortcomings of MSC delivery during *ex situ* dynamic preservation. In a recent systematic review of preclinical studies, we examined the efficacy of EV-therapy derived from stem cells in mitigating IRI in transplantable organs. Our findings indicate that EV-therapy significantly enhances post-reperfusion outcomes, histology, and function in the heart, lung, liver, and kidney, regardless of the originating stem cell source [33]. As EV and soluble factors are unaffected by the “device barrier” phenomenon [28–30], it can be hypothesized that the EV delivery during MP will be more efficient than MSC delivery (Figure 1). Furthermore, whereas MSC suspended in the perfusate at the end of MP are flushed out of the organ before transplantation, the intracellular localization of EV during MP [30, 31] ensures that they will be readily available at the time of graft reperfusion. The EV intracellular localization also prevents their elimination by resident monocytes, and the absence of human leukocytes antigens on EV membranes minimizes the risk of allo-sensitization in the recipient. Additionally, cell-free therapy during MP eliminates the risk of malignant transformation of engrafted cells. Lastly, the use of concentrated EV may offer a selective advantage because they transfer mRNA and miRNA. This transfer has the potential to induce long-lasting biological changes in target cells, which may persist even after graft reperfusion. For these reasons, and because EV possess biological properties comparable to those of the parental stem cell population, it can be hypothesized that EV-therapy will be safer and more efficient than MSC delivery during MP in harnessing MSC properties for repairing and regenerating organs before transplantation. Treatment with EV during MP has already delivered encouraging preliminary results [29, 31]; nevertheless, this hypothesis must be tested in preclinical transplant models of high translational value, as well as in clinical studies.

To move toward clinical applications, it is crucial to determine whether the therapeutic effects of EV match those of their parent stem cells. Preliminary studies suggest that MSC and EV-therapy during MP yield similar results [14, 34]. Nonetheless, further research is required to validate this hypothesis. Additionally, the mechanisms of protection against organ IRI of soluble factors and EV released by different stem cell types should be thoroughly assessed and compared. Indeed, given the complex pathophysiology of IRI, a combined treatment with soluble factors and EV from multiple sources may deliver superior benefits. Dose-finding studies in a clinically relevant model are also necessary to identify the optimal dose of EV and/or soluble factors needed to yield relevant and durable therapeutic effects [33]. Currently, the dose of EV necessary for treating human organs can only be projected based on small animal studies, and inter-species difference may lead to overestimation of the therapeutic dose. This is a crucial point since the large scale production of purified EV is currently an unmet need and one of the major impediments to the clinical application of EV-therapy due to technological limitation. Pre-clinical studies with a larger model, phylogenetically closer to the humans may improve the estimation of the therapeutic dose. Next to organ transplantation, EV-therapy may be of benefit in several medical fields, including genetic and oncological diseases. Indeed, EV can be engineered and programmed to interact with specific cell populations to deliver a cargo enriched with gene modulating and editing agents for the treatment of genetic conditions [35], or chemotherapy and other antineoplastic agents for the treatment of malignant diseases. Thus, there seems to be ample convergence of interests for academic centres and industry to engage in research cooperations to foster technological advancements and develop procedures for scalable production and purification of EV compliant with good manufacturing practice. We strongly advocate for this type of cooperation as an essential step towards bringing EV-therapy to clinical practice, in particular to the novel field of *ex situ* organ repair and regeneration before transplantation.

In conclusion, MSC-therapy during MP is burdened by suboptimal delivery of short-lived MSC. However, their therapeutic benefits may be leveraged using a cell-free therapy consisting of concentrated EV and/or MSC secretome administered during MP. This approach resulted in the intracellular delivery of EV during perfusion and yielded therapeutic benefit in non-transplant models. We hypothesize that technology at nanoscale, such as EV, gene editing, and nanoparticles, have the highest likelihood of successfully translating into clinical applications and will shape the future of *ex situ* organ repair and regeneration before transplantation.

## Abbreviations

EV, extracellular vesicles; IRI, ischemia-reperfusion injury; MAPc, multipotent adult progenitor cells; MP, machine perfusion; MSC, mesenchymal stromal cells; NMP, normothermic machine perfusion.

## References

[1] Hoogduijn M Montserrat N van der Laan L Dazzi F Perico N Kastrup J The Emergence of Regenerative Medicine in Organ Transplantation: 1st European Cell Therapy and Organ Regeneration Section Meeting. Transpl Int (2020) 33(8):833–40. 10.1111/tri.13608 32237237 PMC7497223

[2] Eshmuminov D Becker D Bautista Borrego L Hefti M Schuler MJ Hagedorn C An Integrated Perfusion Machine Preserves Injured Human Livers for 1 Week. Nat Biotechnol (2020) 38(2):189–98. 10.1038/s41587-019-0374-x 31932726 PMC7008032

[3] Martins PN Del Turco S Gilbo N. Organ Therapeutics During Ex-Situ Dynamic Preservation. A Look Into the Future. Eur J Transplant (2022) 1(1):63–78. 10.57603/EJT-010

[4] Rowart P Erpicum P Detry O Weekers L Grégoire C Lechanteur C Mesenchymal Stromal Cell Therapy in Ischemia/Reperfusion Injury. J Immunol Res (2015) 2015:602597–8. 10.1155/2015/602597 26258151 PMC4518154

[5] Eggenhofer E Luk F Dahlke MH Hoogduijn MJ. The Life and Fate of Mesenchymal Stem Cells. Front Immunol (2014) 5:148. 10.3389/fimmu.2014.00148 24904568 PMC4032901

[6] Vandermeulen M Mohamed-Wais M Erpicum P Delbouille MH Lechanteur C Briquet A Infusion of Allogeneic Mesenchymal Stromal Cells After Liver Transplantation: A 5-Year Follow-Up. Liver Transplant (2022) 28(4):636–46. 10.1002/lt.26323 34605167

[7] Podestà MA Remuzzi G Casiraghi F. Mesenchymal Stromal Cell Therapy in Solid Organ Transplantation. Front Immunol (2021) 11. 10.3389/fimmu.2020.618243 PMC7902912 33643298

[8] Casiraghi F Azzollini N Todeschini M Cavinato RA Cassis P Solini S Localization of Mesenchymal Stromal Cells Dictates Their Immune or Proinflammatory Effects in Kidney Transplantation. Am J Transplant (2012) 12(9):2373–83. 10.1111/j.1600-6143.2012.04115.x 22642544

[9] Perico N Casiraghi F Introna M Gotti E Todeschini M Cavinato RA Autologous Mesenchymal Stromal Cells and Kidney Transplantation: A Pilot Study of Safety and Clinical Feasibility. Clin J Am Soc Nephrol (2011) 6(2):412–22. 10.2215/CJN.04950610 20930086 PMC3052234

[10] Pool M Eertman T Sierra Parraga J 't Hart N Roemeling-van Rhijn M Eijken M Infusing Mesenchymal Stromal Cells into Porcine Kidneys During Normothermic Machine Perfusion: Intact MSCs Can Be Traced and Localised to Glomeruli. Int J Mol Sci (2019) 20(14):3607. 10.3390/ijms20143607 31340593 PMC6678394

[11] Mordant P Nakajima D Kalaf R Iskender I Maahs L Behrens P Mesenchymal Stem Cell Treatment Is Associated With Decreased Perfusate Concentration of Interleukin-8 During Ex Vivo Perfusion of Donor Lungs After 18-Hour Preservation. J Heart Lung Transplant (2016) 35(10):1245–54. 10.1016/j.healun.2016.04.017 27444694

[12] Laing RW Stubblefield S Wallace L Roobrouck VD Bhogal RH Schlegel A The Delivery of Multipotent Adult Progenitor Cells to Extended Criteria Human Donor Livers Using Normothermic Machine Perfusion. Front Immunol (2020) 11:1226. 10.3389/fimmu.2020.01226 32714318 PMC7344318

[13] Verstegen MMA Mezzanotte L Ridwan RY Wang K de Haan J Schurink IJ First Report on Ex Vivo Delivery of Paracrine Active Human Mesenchymal Stromal Cells to Liver Grafts During Machine Perfusion. Transplantation (2020) 104(1):e5–e7. 10.1097/TP.0000000000002986 31609902

[14] Gregorini M Corradetti V Pattonieri EF Rocca C Milanesi S Peloso A Perfusion of Isolated Rat Kidney With Mesenchymal Stromal Cells/Extracellular Vesicles Prevents Ischaemic Injury. J Cell Mol Med (2017) 21(12):3381–93. 10.1111/jcmm.13249 28639291 PMC5706569

[15] Thompson ER Bates L Ibrahim IK Sewpaul A Stenberg B McNeill A Novel Delivery of Cellular Therapy to Reduce Ischemia Reperfusion Injury in Kidney Transplantation. Am J Transplant (2021) 21(4):1402–14. 10.1111/ajt.16100 32506663

[16] Brasile L Henry N Orlando G Stubenitsky B. Potentiating Renal Regeneration Using Mesenchymal Stem Cells. Transplantation (2019) 103(2):307–13. 10.1097/TP.0000000000002455 30234788 PMC6347512

[17] Yang L Cao H Sun D Lin L Zheng WP Shen ZY Normothermic Machine Perfusion Combined With Bone Marrow Mesenchymal Stem Cells Improves the Oxidative Stress Response and Mitochondrial Function in Rat Donation After Circulatory Death Livers. Stem Cell Dev (2020) 29(13):835–52. 10.1089/scd.2019.0301 PMC7336881 32253985

[18] Cao H Wu L Tian X Zheng W Yuan M Li X HO-1/BMMSC Perfusion Using a Normothermic Machine Perfusion System Reduces the Acute Rejection of DCD Liver Transplantation by Regulating NKT Cell Co-Inhibitory Receptors in Rats. Stem Cell Res Ther (2021) 12(1):587. 10.1186/s13287-021-02647-5 34819139 PMC8611848

[19] Sun D Yang L Zheng W Cao H Wu L Song H. Protective Effects of Bone Marrow Mesenchymal Stem Cells (BMMSCS) Combined With Normothermic Machine Perfusion on Liver Grafts Donated After Circulatory Death Via Reducing the Ferroptosis of Hepatocytes. Med Sci Monitor (2021) 27:e930258. 10.12659/MSM.930258 PMC8204680 34112750

[20] Tian X Cao H Wu L Zheng W Yuan M Li X Heme Oxygenase-1-Modified Bone Marrow Mesenchymal Stem Cells Combined With Normothermic Machine Perfusion Repairs Bile Duct Injury in a Rat Model of DCD Liver Transplantation Via Activation of Peribiliary Glands Through the Wnt Pathway. Stem Cell Int (2021) 2021:9935370–17. 10.1155/2021/9935370 PMC8275434 34285696

[21] Sierra Parraga JM Rozenberg K Eijken M Leuvenink HG Hunter J Merino A Effects of Normothermic Machine Perfusion Conditions on Mesenchymal Stromal Cells. Front Immunol (2019) 10:765. 10.3389/fimmu.2019.00765 31024574 PMC6469476

[22] La Francesca S Ting AE Sakamoto J Rhudy J Bonenfant NR Borg ZD Multipotent Adult Progenitor Cells Decrease Cold Ischemic Injury in Ex Vivo Perfused Human Lungs: An Initial Pilot and Feasibility Study. Transpl Res (2014) 3(1):19. 10.1186/2047-1440-3-19 PMC4323223 25671090

[23] Nakajima D Watanabe Y Ohsumi A Pipkin M Chen M Mordant P Mesenchymal Stromal Cell Therapy during Ex Vivo Lung Perfusion Ameliorates Ischemia-Reperfusion Injury in Lung Transplantation. J Heart Lung Transplant (2019) 38(11):1214–23. 10.1016/j.healun.2019.07.006 31474491

[24] Lohmann S Pool MBF Rozenberg KM Keller AK Moers C Møldrup U Mesenchymal Stromal Cell Treatment of Donor Kidneys During Ex Vivo Normothermic Machine Perfusion: A Porcine Renal Autotransplantation Study. Am J Transplant (2021) 21(7):2348–59. 10.1111/ajt.16473 33382194

[25] Casiraghi F Perico N Cortinovis M Remuzzi G. Mesenchymal Stromal Cells in Renal Transplantation: Opportunities and Challenges. Nat Rev Nephrol (2016) 12(4):241–53. 10.1038/nrneph.2016.7 26853275

[26] Pool MBF Vos J Eijken M van Pel M Reinders MEJ Ploeg RJ Treating Ischemically Damaged Porcine Kidneys With Human Bone Marrow- and Adipose Tissue-Derived Mesenchymal Stromal Cells During Ex Vivo Normothermic Machine Perfusion. Stem Cell Dev (2020) 29(20):1320–30. 10.1089/scd.2020.0024 32772797

[27] Tetta C Ghigo E Silengo L Deregibus MC Camussi G. Extracellular Vesicles as an Emerging Mechanism of Cell-To-Cell Communication. Endocrine (2013) 44(1):11–9. 10.1007/s12020-012-9839-0 23203002 PMC3726927

[28] Lonati C Bassani GA Brambilla D Leonardi P Carlin A Maggioni M Mesenchymal Stem Cell–Derived Extracellular Vesicles Improve the Molecular Phenotype of Isolated Rat Lungs During Ischemia/Reperfusion Injury. J Heart Lung Transplant (2019) 38(12):1306–16. 10.1016/j.healun.2019.08.016 31530458

[29] Rigo F De Stefano N Navarro-Tableros V David E Rizza G Catalano G Extracellular Vesicles From Human Liver Stem Cells Reduce Injury in an Ex Vivo Normothermic Hypoxic Rat Liver Perfusion Model. Transplantation (2018) 102(5):e205–10. 10.1097/TP.0000000000002123 29424767

[30] De Stefano N Navarro-Tableros V Roggio D Calleri A Rigo F David E Human Liver Stem Cell-Derived Extracellular Vesicles Reduce Injury in a Model of Normothermic Machine Perfusion of Rat Livers Previously Exposed to a Prolonged Warm Ischemia. Transpl Int (2021) 34(9):1607–17. 10.1111/tri.13980 34448268 PMC9291857

[31] Gennai S Monsel A Hao Q Park J Matthay MA Lee JW. Microvesicles Derived From Human Mesenchymal Stem Cells Restore Alveolar Fluid Clearance in Human Lungs Rejected for Transplantation. Am J Transpl (2015) 15(9):2404–12. 10.1111/ajt.13271 PMC4792255 25847030

[32] Rampino T Gregorini M Germinario G Pattonieri EF Erasmi F Grignano MA Extracellular Vesicles Derived From Mesenchymal Stromal Cells Delivered During Hypothermic Oxygenated Machine Perfusion Repair Ischemic/Reperfusion Damage of Kidneys From Extended Criteria Donors. Biology (Basel) (2022) 11(3):350. 10.3390/biology11030350 35336724 PMC8945029

[33] Blondeel J Gilbo N De Bondt S Monbaliu D. Stem Cell Derived Extracellular Vesicles to Alleviate Ischemia-Reperfusion Injury of Transplantable Organs. A Systematic Review. Stem Cell Rev Rep. (2023) 19:2225–50. 10.1007/s12015-023-10573-7 37548807

[34] Stone ML Zhao Y Robert Smith J Weiss ML Kron IL Laubach VE Mesenchymal Stromal Cell-Derived Extracellular Vesicles Attenuate Lung Ischemia-Reperfusion Injury and Enhance Reconditioning of Donor Lungs After Circulatory Death. Respir Res (2017) 18(1):212. 10.1186/s12931-017-0704-9 29268735 PMC5740880

[35] Herrera Sanchez MB Previdi S Bruno S Fonsato V Deregibus MC Kholia S Extracellular Vesicles From Human Liver Stem Cells Restore Argininosuccinate Synthase Deficiency. Stem Cell Res Ther (2017) 8(1):176. 10.1186/s13287-017-0628-9 28750687 PMC5531104

[36] McAuley DF Curley GF Hamid UI Laffey JG Abbott J McKenna DH Clinical Grade Allogeneic Human Mesenchymal Stem Cells Restore Alveolar Fluid Clearance in Human Lungs Rejected for Transplantation. Am J Physiology-Lung Cell Mol Physiol (2014) 306(9):L809–L815. 10.1152/ajplung.00358.2013 PMC4010648 24532289

[37] Martens A Ordies S Vanaudenaerde BM Verleden SE Vos R Van Raemdonck DE Immunoregulatory Effects of Multipotent Adult Progenitor Cells in a Porcine Ex Vivo Lung Perfusion Model. Stem Cell Res Ther (2017) 8(1):159. 10.1186/s13287-017-0603-5 28676074 PMC5497348

[38] Sasajima H Miyagi S Kakizaki Y Kamei T Unno M Satomi S Cytoprotective Effects of Mesenchymal Stem Cells During Liver Transplantation From Donors After Cardiac Death in Rats. Transpl Proc (2018) 50(9):2815–20. 10.1016/j.transproceed.2018.02.180 30401403

[39] Yang L Cao H Sun D Hou B Lin L Shen ZY Bone Marrow Mesenchymal Stem Cells Combine With Normothermic Machine Perfusion to Improve Rat Donor Liver Quality—The Important Role of Hepatic Microcirculation in Donation After Circulatory Death. Cell Tissue Res (2020) 381(2):239–54. 10.1007/s00441-020-03202-z 32347385 PMC7369267

