# Supplementary material for: Organ Repair and Regeneration During Ex Situ Dynamic Preservation: The Future is Nano
Source: Transpl Int. 2023 Nov 10;36:11947. doi: 10.3389/ti.2023.11947 (PMC10667440; doi:10.3389/ti.2023.11947)
Supplement: Supplementary file 1 [file DataSheet1.docx]

# Supplementary Information

Supplementary Methods.

To scope the available evidence on mesenchymal stromal cells (MSC) therapy delivery during machine perfusion (MP) prior to transplantation, a systematic search of Medline (PubMed) database was performed on April 1, 2022. A research strategy was built including medical subject headings (MeSH) terms and relevant free-text synonyms for the key concepts ‘stem cells’, ‘organ transplantation’, and ‘machine perfusion’ (Appendix S1). Titles and abstracts were screened to identify potentially relevant studies. Next, the full texts of the selected items were screened for eligibility. Peer-reviewed studies reporting on MSCs therapy during MP were considered eligible regardless of organ perfused and type of MSCs utilized. Review articles, letter to the editor, and publication with no full text available or published in languages other than English were excluded. Finally, the reference list of every article was searched for relevant titles that could not be identified with the primary search.

The primary search originated 76 items. No additional papers were retrieved after searching the reference lists for relevant titles. After title and abstract screening, 36 articles were excluded because unrelated to the searched topic, while additional 17 items were excluded based on article type and language. After screening 23 full texts, 21 manuscripts were considered eligible (Figure S1). Data regarding the type of organ, type and duration of MP, type and dose of MSCs, as well as whether they were paracrine active, migrated out of the vascular lumen and engrafted in the parenchyma, and their effect during perfusion and/or after transplantation were extracted from these 21 manuscripts to facilitate the formulation of opinions based on experimental results. A summary of the data extracted is reported in Table 1 of the main manuscript.

Supplementary Figure S1: Flow diagram summarizing literature search and selection of papers.

53 records excluded

- 36 unrelated topic
- 14 article type
- 3 language

Records screened
(n = 76)

## Identification

## Eligibility

## Included

## Screening

Studies included for data extraction
(n = 21)

Full-text articles assessed for eligibility
(n = 23)

Records identified through database searching (Medline)
(n = 76)

2 records excluded

- 1 study did not include a machine perfusion group
- 1 study did not use MSC

Appendix S1: research strategy

The following research strategy was built to search the Medline (PubMed) database on April 1, 2022.

**Concept 1: stem cells.**

“stem cells”[Mesh] OR “mesenchymal stem cells”[Mesh] OR “induced pluripotent stem cells”[Mesh] OR “adult stem cells”[Mesh] OR “fetal stem cells”[Mesh] OR “embryonic stem cells”[Mesh] OR “pluripotent stem cells”[Mesh] Or “multipotent stem cells”[Mesh] OR “totipotent stem cells”[Mesh] OR “hematopoietic stem cells”[Mesh] OR “oogonal stem cells”[Mesh] OR “adult germline stem cells”[Mesh] OR “peripheral blood stem cells”[Mesh] OR “human embryonic stem cells”[Mesh] OR “mouse embryonic stem cells”[Mesh] OR “stem cells”[tiab] OR “stem cell*”[tiab] OR “precursor cells”[tiab] OR “precursor cell*”[tiab] OR “precursors”[tiab] OR “precursor*”[tiab] OR “progenitor cells”[tiab] OR “progenitor cell*”[tiab] OR “progenitors”[tiab] OR “progenitor*”[tiab]

**Concept 2: organ transplantation.**

“organ transplantation”[Mesh] OR “transplants”[Mesh] OR “allograft”[Mesh] OR “graft enhancement”[Mesh]

**Concept 3: machine perfusion.**

“organ preservation”[Mesh] OR “perfusion”[Mesh] OR “machine perfusion”[tiab] OR “isolated perfusion”[tiab] OR “ex vivo perfusion”[tiab] OR “ex situ perfusion”[tiab]

**Final research strategy.**

(“stem cells”[Mesh] OR “mesenchymal stem cells”[Mesh] OR “induced pluripotent stem cells”[Mesh] OR “adult stem cells”[Mesh] OR “fetal stem cells”[Mesh] OR “embryonic stem cells”[Mesh] OR “pluripotent stem cells”[Mesh] Or “multipotent stem cells”[Mesh] OR “totipotent stem cells”[Mesh] OR “hematopoietic stem cells”[Mesh] OR “oogonal stem cells”[Mesh] OR “adult germline stem cells”[Mesh] OR “peripheral blood stem cells”[Mesh] OR “human embryonic stem cells”[Mesh] OR “mouse embryonic stem cells”[Mesh] OR “stem cells”[tiab] OR “stem cell*”[tiab] OR “precursor cells”[tiab] OR “precursor cell*”[tiab] OR “precursors”[tiab] OR “precursor*”[tiab] OR “progenitor cells”[tiab] OR “progenitor cell*”[tiab] OR “progenitors”[tiab] OR “progenitor*”[tiab]) AND (“organ transplantation”[Mesh] OR “transplants”[Mesh] OR “allograft”[Mesh] OR “graft enhancement”[Mesh]) AND (“organ preservation”[Mesh] OR “perfusion”[Mesh] OR “machine perfusion”[tiab] OR “isolated perfusion”[tiab] OR “ex vivo perfusion”[tiab] OR “ex situ perfusion”[tiab]).
